# Salivary cortisol measurements in mechanically ventilated patients

**DOI:** 10.1186/cc9829

**Published:** 2011-03-11

**Authors:** D Vassiliadi, I Mavrou, M Tzanela, N Nikitas, M Theodorakopoulou, S Orfanos, A Armaganidis, S Tsagarakis, I Dimopoulou

**Affiliations:** 1 Attikon University Hospital, Athens, Greece; 2 Evagelismos Hospital, Athens, Greece; 3 Alexandra Hospital, Athens, Greece; 4 Polyclinic Hospital, Athens, Greece

## Introduction

Salivary cortisol is a reliable tool to evaluate the normal or disordered control of the hypothalamic-pituitary-adrenal (HPA) axis. Despite this, salivary cortisol has been rarely assessed in the setting of intubated, critically ill patients. The purpose of the current study was to investigate the utility of salivary cortisol measurements in an intensive care population.

## Methods

Thirty-nine (25 men) consecutive, critically ill patients with a mean (± SD) age of 65 ± 22 years having various illnesses were included in the present study. Sixteen patients had sepsis. Mean APACHE II and SOFA scores were 17 ± 10 and 7 ± 3, respectively. Mean albumin was 3.0 ± 0.7 g/dl. Within 48 hours of ICU admission, morning cosyntropin stimulation tests (250 μg, i.v.) were performed. Serum total cortisol and salivary cortisol were measured before and 30 minutes after consyntropin administration. In eight healthy controls, baseline salivary cortisol was also measured.

## Results

Patients had higher baseline salivary cortisol than healthy controls (1.13 ± 0.80 μg/dl vs. 0.33 ± 0.80 μg/dl, *P *= 0.002). Baseline and cosyntropin-stimulated serum total cortisol were 21 ± 11 μg/dl and 31 ± 13 μg/dl, respectively (*P *< 0.001). Baseline and cosyntropin-stimulated salivary cortisol were 1.13 ± 0.80 μg/dl and 1.4 ± 0.90 μg/dl, respectively (*P *= 0.004). Baseline serum total cortisol correlated with baseline salivary cortisol in patients with albumin values >2.5 g/dl (*r *= 0.60, *P *= 0.01). In contrast, there was no correlation between these variables in patients having albumin concentrations ≤2.5 g/dl. Stimulated serum total cortisol did not correlate with stimulated salivary cortisol in either of the two subgroups.

## Conclusions

Salivary cortisol measurement is easy to obtain in critically ill patients. Salivary cortisol is higher compared with healthy controls and increases significantly following stimulation with cosyntropin. Whether salivary cortisol is superior to serum total cortisol measurements in the assessment of the HPA axis activity requires further investigation.

